# Estimated glucose disposal rate is associated with retinopathy and kidney disease in young people with type 1 diabetes: a nationwide observational study

**DOI:** 10.1186/s12933-023-01791-x

**Published:** 2023-03-19

**Authors:** Wedén Linn, Martina Persson, Björn Rathsman, Johnny Ludvigsson, Marcus Lind, Mikael Andersson Franko, Thomas Nyström

**Affiliations:** 1 Department of Clinical Science and Education, Karolinska Institutet, Södersjukhuset, Sjukhusbacken 10, 118 83 Stockholm, Sweden; 2 Sachs’ Children and Youth Hospital, Södersjukhuset, Stockholm, Sweden; 3 Division of Paediatrics, Department of Biomedical and Clinical Sciences, Linköping University, Linköping, Sweden; 4 Crown Princess Victoria Children’s Hospital, Region Östergötland, Linköping, Sweden; 5 Department of Medicine, NU Hospital Group, Uddevalla, Sweden; 6 Department of Molecular and Clinical Medicine, Institute of Medicine, University of Gothenburg, Gothenburg, Sweden

**Keywords:** Albuminuria, Estimated glucose disposal rate (eGDR), Insulin resistance, Retinopathy, Kidney disease, Type 1 diabetes

## Abstract

**Aims:**

The aim of this study was to investigate the association between estimated glucose disposal rate (eGDR), a proxy for insulin resistance, and retinopathy or kidney disease, i.e. micro-, or macroalbuminuria, in young individuals with type 1 diabetes (T1D).

**Material and Methods:**

Using data from the Swedish pediatric registry for diabetes (SweDiabKids) and the registry for adults (NDR), all individuals with T1D with a duration of diabetes of less than 10 years between 1998 and 2017 were included. We calculated the crude incidence rates with 95% confidence intervals (CIs) and used multivariable Cox regression to estimate crude and adjusted hazard ratios (HRs) for two cohorts: retinopathy cohort or kidney disease cohort, stratified by eGDR categories: < 4, 4 to 5.99, 6 to 7.99, and ≥ 8 mg/kg/min (reference).

**Results:**

A total of 22 146 (10 289 retinopathy cohort, and 11 857 kidney disease cohort with an overlapping of 9575) children and adults with T1D (median age 21 years, female 42% and diabetes duration of 6 and 7 years, respectively for the cohorts) were studied. During a median follow-up of 4.8 years (IQR 2.6–7.7) there were 5040 (24.7%), 1909 (48.1%), 504 (52.3%) and 179 (57.6%) events for retinopathy in individuals with an eGDR ≥ 8, 7.99 to 6, 5.99 to 4, and < 4 mg/kg/min, respectively. Corresponding numbers for kidney disease was 1321 (6.5%), 526 (13.3%), 255 (26.8%) and 145 (46.6%). After multiple adjustments for different covariates, individuals with an eGDR 7.99 to 6, 5.99 to 4 and < 4 mg/kg/min, had an increased risk of retinopathy compared to those with an eGDR ≥ 8 mg/kg/min (adjusted HRs, 95% CIs) 1.29 (1.20 to 1.40); 1.50 (1.31 to 1.71) and 1.74 (1.41 to 2.14). Corresponding numbers for kidney disease was (adjusted HRs, 95% CIs) 1.30 (1.11 to 1.52); 1.58 (1.25 to 1.99) and 1.33 (0.95 to 1.86), respectively.

**Conclusions:**

eGDR, a proxy for insulin resistance, is associated with retinopathy and kidney disease in young adults with T1D. The risk of retinopathy increased with lower eGDR. The risk of kidney disease also increased with lower eGDR; however results show no association between the lowest eGDR and kidney disease. eGDR can be helpful to identify young T1D individuals at risk.

**Supplementary Information:**

The online version contains supplementary material available at 10.1186/s12933-023-01791-x.

## Introduction

Individuals with type 1 diabetes (T1D) are at increased risk of macrovascular and cardiac complications, in which microvascular complications may predict the risk [1]. Intensified glycemic control can lower the risk [2, 3]. However, in spite of good glycemic control individuals with T1D are still at higher risk of cardiovascular complications compared to individuals without T1D [4]. This may be explained by other cardiovascular risk factors besides hyperglycemia such as dyslipidemia, kidney disease and hypertension [5].

The term double diabetes, first described in 1991, was initially defined as an individual with T1D with a family history of type 2 diabetes (T2D) [6]. Today, it lacks a clear definition but is often referred to as T1D with components of the metabolic syndrome, which is typically observed in people with T2D. Individuals with double diabetes often have poorer glycemic control with increased insulin requirements, and components of the metabolic syndrome such as dyslipidemia, insulin resistance, obesity and hypertension [7, 8]. Moreover, double diabetes is linked to both genetic and life-style factors, e.g., poor physical activity, repeated hypoglycemia and peripheral insulin resistance [7–9].

The gold standard for measuring insulin resistance is the euglycemic hyperinsulinemic clamp technique [10]. Since this method is invasive and time consuming it is not suitable for daily clinical practice or larger studies. Estimated glucose disposal rate (eGDR) using standard clinical measures (hemoglobin glycated A1c [HbA1c], hypertension and waist circumference, or body mass index [BMI]) is derived from clamps in young people with T1D, and has been shown to correlate well [11]. On the other hand, independent cohorts trying to validate the above eGDR formula [11] have not come to the same conclusion [12–14]. Although, the eGDR has been shown to be good marker of increased risk for different outcomes [15–17], it is not entirely sure that it reflects insulin resistance. Several studies among individuals with T1D have shown a connection between double diabetes and macrovascular complications independent of glycemic control [18–20]. Recently, in a large nationwide cohort study among individuals with T1D, our group demonstrated a strong association between eGDR and risk of preterm mortality [16].

Not only macrovascular complications are associated with insulin resistance, but also microvascular complications [15]. There is a limited number of large studies that have examined the association between insulin resistance and microvascular complications among young individuals with T1D [17, 21–24]. The aim of this study is to investigate whether eGDR, as a proxy for insulin resistance, is associated with increased risk of retinopathy and kidney disease, i.e. microalbuminuria, or macroalbuminuria, in young people with T1D.

## Methods

### Study design and study population

The study was a nationwide, observational cohort study. The study was approved by the Swedish Ethical Review Authority (Dnr 977-17).

We used data from the Swedish national diabetes registry for adults (NDR) and the Swedish pediatric registry for diabetes (SweDiabKids), no other registers were used. Participants provided informed consent when entering the registries. NDR defines T1D on the basis of epidemiological data: treatment with insulin and a diagnosis at the age of 30 years or younger, which has shown to be validated in 97% of cases [25]. In children and adolescents, HLA genotype and autoantibodies are determined before diagnosis. The majority of all Swedish individuals with T1D are registered in NDR, or SweDiabKids. Data was extracted from NDR from 1998, and from SweDiabKids from 2000, both until 31^st^ December 2017. Between these time points all children, adolescents and adults in the registries were included if they had a diagnosis of T1D since ten years, or less when first recorded in the registries. The age span in the whole cohort was 0–39 years, i.e., patients were included if they are diagnosed with T1D before 30 years of age and if duration was < 10 years.

Data extracted from the registers were age, sex, diabetes duration, weight, height, BMI, blood pressure, HbA1c, total cholesterol, high-density lipoprotein (HDL), low-density lipoprotein (LDL), triglycerides, smoking, physical activity, type of diabetes treatment, micro/macroalbuminuria, estimated glomerular filtration rate (eGFR), and result of retinopathy screening. Categorization of the variables from the registers are shown in Tables 1 and 2, also described below (endpoints).

**Table 1:** Baseline characteristics of all patients divided in the retinopathy cohort and kidney disease cohort, respectively

	Retinopathy analysis	Kidney disease analysis
Number	10,289	11,857
Age, yrs	21 (19–26)	21 (19–26)
Males	58.1%	57.3%
Debut age, yrs	16 (11–21)	15 (10–21)
0–10 yrs	18.3%	22.9%
10–15 yrs	26.3%	25.7%
15–20 yrs	23.7%	21.9%
20–25 yrs	18.9%	17.6%
25–30 yrs	12.7%	11.9%
Duration, yrs	6 (3–9)	7 (3–10)
Follow-up time, yrs	4.8 (2.6–7.7)	5.4 (2.9–8.7)
eGDR measurements per individual	4 (2–6)	4 (2–7)
eGDR, mg/kg/min	9.0 (8.0–9.8)	8.9 (8.0–9.7)
< 4	1.7%	1.5%
4 ≤ to < 6	5.1%	5.0%
6 ≤ to < 8	18.5%	19.4%
≥ 8	74.8%	74.1%
LDL-cholesterol, mmol/L	2.43 (1.97–2.96)	2.43 (1.98–2.98)
< 2.6	59.1%	58.6%
2.6 ≤ to < 3.4	28.3%	28.3%
3.4 ≤ to < 4.1	8.9%	9.3%
≥ 4.1	3.6%	3.8%
HDL-cholesterol, mmol/L	1.4 (1.2–1.7)	1.4 (1.2–1.7)
< 1.1	13.1%	13.2%
≥ 1.1	86.9%	86.8%
Total-cholesterol, mmol/L	4.4 (3.8–5.0)	4.4 (3.8–5.0)
< 4.5	54.5%	53.9%
≥ 4.5	45.5%	46.1%
Triglyceride, mmol/L	0.90 (0.62–1.28)	0.90 (0.66–1.30)
< 1.7	86.5%	86.1%
≥ 1.7	13.5%	13.9%
HbA1c, mmol/mol	60 (51–71)	61 (52–72)
< 48	17.2%	16.4%
48 ≤ to < 58	24.9%	23.8%
58 ≤ to < 70	29.7%	30.5%
≥ 70	28.2%	29.2%
BMI (Kg/m^2^)^*^	23.6 (21.6–26.2)	23.4 (21.3–26.1)
Normal	64.5%	65.4%
Overweight	26.3%	25.7%
Obese	9.2%	8.9%
Smokers	11.0%	11.3%
Physical activity; daily	18.2%	17.6%
Physical activity; 3–5 times/week	33.2%	33.6%
Physical activity; 1–2 times/week	26.2%	26.9%
Physical activity; < 1 times/week	14.1%	13.7%
Physical activity; never	8.3%	8.1%
Insulin method; injection	81.3%	79.5%
Insulin method; pump	18.7%	20.5%
ASA; yes	0.8%	0.7%
Antihypertensive therapy; yes	3.5%	3.0%
Lipid lowering drug; yes	3.0%	2.6%
Hypertension	5.2%	5.0%
eGFR, mL/min	123 (106–145)	124 (107–147)
< 30	0.06%	0.03%
30 ≤ to < 45	0.03%	0.02%
45 ≤ to < 60	0.13%	0.10%
60 ≤ to < 90	7.26%	6.79%
≥ 90	92.53%	93.06%

*ASA* Acetylsalicylic acid, *BMI* Body mass index, *eGFR* Estimated glomerular filtration rate, *HbA1c* Glycated hemoglobin 1c, *HDL-Cholesterol* High-density lipoprotein-Cholesterol, *LDL-Cholesterol* Low-density lipoprotein-Cholesterol. *Yrs* years

^*^isoBMI was used in individuals < 18 yrs

**Table 2:** All categorized covariates that were multivariate adjusted for in the final model

	Retinopathy			Kidney disease		
	Hazard ratio	Confidence interval	p-value	Hazard ratio	Confidence interval	p-value
8 ≤ eGDR, mg/kg/min	REF			REF		
6 ≤ eGDR < 8, mg/kg/min	1.29	1.20–1.40	< 0.001	1.30	1.11–1.51	0.001
4 ≤ eGDR < 6, mg/kg/min	1.50	1.31–1.71	< 0.001	1.58	1.25–1.99	< 0.001
eGDR < 4, mg/kg/min	1.74	1.41–2.14	< 0.001	1.33	0.95–1.86	0.099
Male	REF			REF		
Female	0.99	0.92–1.06	0.70	1.34	1.17–1.53	< 0.001
Age at diabetes onset < 15 yrs	REF					
15 ≤ Age < 20 yrs	2.27	1.63–3.15	< 0.001	3.88	2.79–5.40	< 0.001
20 ≤ Age < 25 yrs	3.62	2.62–5.00	< 0.001	4.09	2.95–5.66	< 0.001
25 ≤ Age < 30 yrs	3.91	2.82–5.42	< 0.001	3.04	2.15–4.29	< 0.001
30 ≤ Age < 40 yrs	3.62	2.59–5.05	< 0.001	3.49	2.44–5.01	< 0.001
Physical activity, daily	REF			REF		
Physical activity, 3–5 times/week	1.03	0.93–1.13	0.59	0.78	0.64–0.94	0.009
Physical activity, 1–2 times/week	1.01	0.91–1.11	0.88	1.04	0.86–1.25	0.70
Physical activity, < 1 times/week	1.10	0.98–1.23	0.091	1.11	0.90–1.37	0.34
Physical activity, never	1.01	0.88–1.15	0.93	1.37	1.08–1.72	0.008
Non-smoker	REF			REF		
Smoker	1.31	1.19–1.44	< 0.001	1.24	1.04–1.48	0.014
Insulin method, injection	REF			REF		
Insulin method, pump	0.98	0.91–1.06	0.66	0.96	0.82–1.12	0.61
LDL < 2.6, mmol/L	REF			REF		
2.6 ≤ LDL < 4.1, mmol/L	1.09	1.00–1.18	0.062	1.21	1.02–1.44	0.027
4.1 ≤ LDL, mmol/L	1.14	0.96–1.36	0.14	1.36	1.01–1.83	0.045
HDL < 1.1, mmol/L	REF			REF		
1.1 ≤ HDL, mmol/L	0.94	0.85–1.04	0.21	0.89	0.74–1.07	0.23
Cholesterol < 4.5, mmol/L	REF			REF		
Cholesterol ≥ 4.5, mmol/L	0.92	0.84–1.00	0.63	0.97	0.81–1.16	0.74
Triglyceride < 1.7, mmol/L	REF			REF		
1.7 ≤ Triglyceride, mmol/L	1.21	1.10–1.34	< 0.001	1.43	1.21–1.69	< 0.001
ASA, no	REF			REF		
ASA, yes	1.33	0.99–1.79	0.056	1.71	1.12–2.60	0.012
Antihypertensive, no	REF			REF		
Antihypertensive therapy, yes	0.85	0.72–1.00	0.052	3.96	3.15–4.98	< 0.001
Lipid lowering drug, no	REF			REF		
Lipid lowering drug, yes	1.13	0.99–1.30	0.078	1.02	0.79–1.32	0.87
90 ≤ eGFR, mL/min	REF			REF		
60 ≤ eGFR < 90, ml/min	0.85	0.75–0.97	0.013	1.02	0.78–1.33	0.88
45 ≤ eGFR < 60, ml/min	0.86	0.38–1.94	0.72	6.66	3.07–14.42	< 0.001
30 ≤ eGFR < 45, ml/min	0.73	0.23–2.32	0.60	9.26	2.92–29.33	< 0.001
eGFR < 30, mL/min	0.79	0.11–5.67	0.82	5.61	1.72–18.30	0.004

All covariates are time-varying, but sex. Continuous variables are presented as medians and interquartile range and categorical variables as proportions

*ASA* acetylsalicylic acid, *BMI* body mass index, *eGDR* estimated glucose disposal rate, *eGFR* estimated glomerular filtration rate, *HbA1c* glycated haemoglobin 1c, *HDL cholesterol* high-density lipoprotein cholesterol, *LDL* cholesterol low-density lipoprotein cholesterol. *Yrs* years

In the final data set individuals with at least one registration of examination of retinopathy, or kidney disease was included. They also needed at least one registration of eGDR (consisting of the variables BMI, HbA1c and hypertension yes/no) and at least one registration of each of the covariates that was used in the final model. In total 15 111 individuals were recruited from SweDiabKids and 19 298 individuals were recruited from NDR. 26 786 individuals had T1D with < 10 years duration when entering the registry. After including patients with at least one examination of retinopathy or nephropathy, one eGDR observation and at least one observation for each covariate, 10 289 cases in the retinopathy cohort and 11 857 cases in the nephropathy cohort remained Figure S1 (Additional file 1). The overlap between cohorts included 9575 individuals.

### Excluded patients

Due to lack of data on retinopathy/kidney disease, eGDR and/or a covariate 16 497 individuals (61%) were excluded from retinopathy analysis (72% from SweDiabKids and 47% from NDR, respectively) and 14 929 individuals (55%) from kidney disease analysis (62% from SweDiabKids and 39% from NDR, respectively) Figure S1 (Additional file 1). Also, individuals before index date with already known retinopathy, or kidney disease were excluded from the study. Baseline characteristics of excluded patients are shown in Table S1 (Additional file 1).

### eGDR procedures and categorization

eGDR_BMI_ (mg/kg/min) was calculated as previously described [11] based on the following formula: eGDR_BMI_ = 19.02 − (0.22*BMI) − (3.26 * HT) − (0.61*HbA1c) (Personal communication Katherine Williams, MD, MPH, Pittsburgh, PA, US). *BMI = body mass index (kg/m*^*2*^*), HT = hypertension (yes = 1/no = 0), and HbA1c = HbA1c (DCCT %)*.

Hypertension was defined as a blood pressure > 140/90 mmHg or current use of any anti-hypertensive agents. For individuals < 18 years old, we used IsoBMI [26]. Analyses of HbA1c levels were performed at certified local laboratories and reported according to the International Federation of Clinical Chemistry standard, measured in mmol/mol. We converted all HbA1c values to standard values according to the National Glycohemoglobin Standardization Program [27].

Based on previous studies [16, 23], we categorized individuals with T1D into four groups according to eGDR levels as follows: < 4, 4 to 5.99, 6 to 7.99, and ≥ 8 mg/kg/min. The highest eGDR category (≥ 8 mg/kg/min) was used as the reference category. The individual components of eGDR were assessed using updated means. During the follow up, study participants could change category if their eGDR worsened, or improved during the study.

### Endpoints and index date

Endpoints of retinopathy were classified as any retinopathy, preproliferative diabetic retinopathy (PPDR), or proliferative diabetic retinopathy (PDR). Any retinopathy included any signs of retinopathy, i.e. simplex retinopathy, PPDR or PDR. PDR was defined as evidence of current proliferations or any earlier laser photocoagulation. Endpoints of kidney disease were classified as any microalbuminuria defined as two positive test results from three samples taken within one year with an albumin/creatinine ratio of 3–30 mg mmol^−1^, or urinary albumin of 20–200 µg min^−1^ (20–300 mg/L), or macroalbuminuria defined as an albumin/creatinine ratio > 30 mg mmol^−1^, or urinary albumin > 200 µg min^−1^ (> 300 mg L^−1^) [3].

All individuals were followed from the first observation (between 1998 and 2017) when they first appeared in the register, i.e. index date, until the first event of any retinopathy, or any kidney disease (microalbuminuria/macroalbuminuria), or until the end of the study 31^st^ of December 2017, whichever came first. Follow up did not end if mild retinopathy, i.e. simplex retinopathy, or microalbuminuria was registered. If more severe forms were registered, milder forms were ignored, and the first diagnosis of the severe form was included.

### Statistical analysis

Baseline characteristics (clinical data) are based on the first available observation (between 1998 and 2017) from index date and after that with no specific time limit, for each individual in the study. Continuous variables are presented as medians with interquartile range (IQR), due to the contribution of data, and categorical variables as proportions. Crude and adjusted hazard ratios (HRs) and confidence intervals (CIs) were estimated using univariable and multivariable Cox regression models with time until endpoints, that is retinopathy or kidney disease, as response variables and time-varying covariates as exposure variables, that is eGDR, and potential confounders. Exposure to risk of retinopathy and kidney disease starts at diabetes onset, but follow-up starts at first eGDR observation. This means that data are left-truncated as well as right-censored handled accordingly.

Instead, age at diabetes onset was included as a covariate. All covariates which were adjusted for in the final model were time-varying, but sex. The time-varying variables, i.e. numbers and timespan per patient are shown in Table S2 (Additional file 1), and were calculated as follows: at each retinopathy or nephropathy examination, numerical variables were calculated as the mean of each registered value since the last examination or start of the study period. If no values were available during this interval, the value from the previous interval was used. For categorical variables, the mode was used rather than the mean. When no observations of a covariate had been made, the value at the previous examination was used. We did not replace missing values by multiple imputation but chose to exclude patients with missing values.

In the calculation of eGDR, the most recent value for each variable in the formula was used with no time limit. This means that eGDR could be based on variables that were collected at different time points. This was done to increase the number of non-missing eGDR values. As new values were registered, eGDR was continually updated mean, thereby making it possible for individuals to change category over time to reflect the varying insulin resistance.

Since retinopathy and kidney disease most often is symptom-free, these conditions can only be discovered at an examination. This means that the outcome is interval censored; that is, the exact time point of the outcome can occur anywhere between a negative and a positive examination. It was assumed that a milder complication precedes a serious complication even if not registered. Cox regression for interval censored time-to-event is not well defined so we used the following algorithm. First, we simulated 1000 data sets where the survival times were uniformly sampled between the time point of the last negative examination and the time point of the first positive examination. Then, Cox regressions were carried out on all data sets and coefficients and standard errors were summarized to account for both estimation error in each model and censoring error between models [28].

Estimated cumulative risks of retinopathy and kidney disease, respectively, stratified over eGDR categories were calculated using the Kaplan–Meier estimator adjusted for left truncated and interval censored observations [29]. Pointwise 95% confidence intervals were calculated based on estimated variances using Greenwood’s formula [30].

Survival curves are calculated from the simulated data sets and time-varying covariates to reflect the assumptions behind the Cox regressions [31]. Smooth ridge regression was used for the interaction to stabilize results for combinations with few observations.

The relative predictive performance for each variable in the eGDR formula was evaluated using Heller R^2^ for the explained risk in the proportional hazards model [32].

## Results

### Study population and patient characteristics

Baseline characteristics of the study participants in the retinopathy and nephropathy cohorts, respectively, are shown in Table 1, and after stratification into different eGDR categories in Table S3 (Additional file 1). The median age at index date in both cohorts was 21 years. 58% were males in the retinopathy cohort and 57% in the kidney disease cohort. Debut age of T1D was 16 years and 15 years, in the retinopathy and kidney disease cohorts, respectively. The median duration of diabetes before entering the study was 6 years and 7 years, respectively. The median eGDR was 9 mg/kg/min in both cohorts and in both cohorts the distribution of individuals in the different eGDR categories was very similar with a majority (around 74%) in the reference category eGDR ≥ 8 mg/kg/min. In both cohorts median HbA1c was approximately 60 mmol/mol, and median BMI was 23 kg/m^2^. 26% of individuals were overweight (BMI > 25 kg/m^2^) and 9% were obese (BMI > 30 kg/m^2^), both cohorts. Finally, 5% of individuals had hypertension. All covariates, multivariable adjusted for in the final model, are shown in Table 2.

### eGDR and retinopathy

**Fig. 1:**
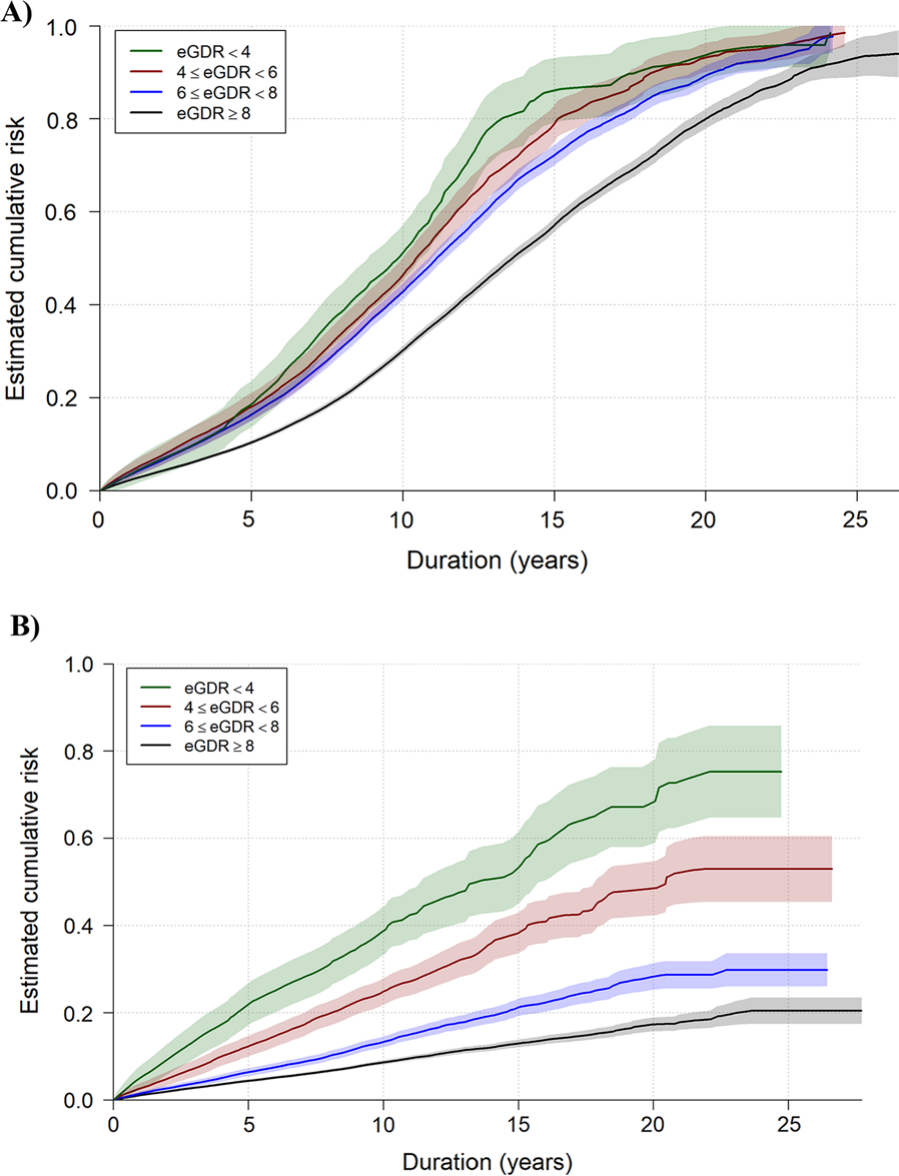
Estimated crude cumulative risk curves illustrated the accumulated estimated risk of retinopathy (**A**) and kidney disease (**B**) based on these observed time intervals in young people with type 1 diabetes (eGDR = estimated glucose disposal rate). The shaded are represents the 95% confidence interval of the estimated crude curves

The estimated retinopathy crude cumulative risk curves illustrating the accumulated risk for retinopathy are shown in Fig. 1A. Median follow up time for the retinopathy cohort was 4.8 (IQR 2.6–7.7) years. The event rate of any retinopathy in each eGDR categories, and the relative risks (HRs) between eGDR and retinopathy are shown in Table 3. After adjustment for age and sex, and all covariates in Table 1, lower eGDR was associated with an increased risk of retinopathy (Table 3). The presence of kidney disease was not significant (P = 0.21) when included as a covariate, indicating no competing risk between retinopathy and kidney disease (Additional file 1: Table S4).

**Table 3:** Number of events, event rate and relative risks for any retinopathy, i.e. mild to severe retinopathy, and kidney disease, i.e. microalbuminuria or macroalbuminuria, in children and adults with type 1 diabetes

Exposure mg/kg/min	Events n (%)	Event rate 100 person-yrs	Crude HR	HR adjusted for sex and age	HR adjusted
Retinopathy					
eGDR ≥ 8	5040 (24.7)	4.11 (3.99–4.22)	REF	REF	REF
6 ≤ eGDR < 8	1909 (48.1)	7.47 (7.13–7.80)	1.61 (1.53–1.70) < 0.001	1.47 (1.40–1.55) < 0.001	1.29 (1.20–1.40) < 0.001
4 ≤ eGDR < 6	504 (52.3)	8.85 (8.08–9.62)	1.91 (1.74–2.09) < 0.001	1.57 (1.43–1.72) < 0.001	1.50 (1.31–1.71) < 0.001
eGDR < 4	179 (57.6)	9.63 (8.22–11.05)	2.28 (1.96–2.66) < 0.001	1.73 (1.49–2.02) < 0.001	1.74 (1.41–2.14) < 0.001
PPDR/PDR/Laser photocoagulation vs. non/simplex					
eGDR ≥ 8	233 (1.1)	0.17 (0.15–0.19)	REF	REF	REF
6 ≤ eGDR < 8	181 (4.6)	0.57 (0.49–0.66)	2.56 (2.10–3.11) < 0.001	2.33 (1.92–2.84) < 0.001	1.71 (1.32–2.22) < 0.001
4 ≤ eGDR < 6	52 (5.5)	0.73 (0.53–0.92)	3.06 (2.26–4.14) < 0.001	2.52 (1.86–3.42) < 0.001	1.53 (1.00–2.34) 0.051
eGDR < 4	23 (7.4)	1.05 (0.62–1.48)	4.58 (2.98–7.05) < 0.001	3.45 (2.24–5.32) < 0.001	1.66 (0.90–3.07) 0.11
PDR/Laser photocoagulation vs. non/simplex/PPDR					
eGDR ≥ 8	82 (0.4)	0.06 (0.05–0.07)	REF	REF	REF
6 ≤ eGDR < 8	60 (1.5)	0.19 (0.14–0.24)	2.45 (1.75–3.44) < 0.001	2.19 (1.56–3.07) < 0.001	1.45 (0.93–2.25) 0.10
4 ≤ eGDR < 6	21 (2.2)	0.29 (0.17–0.41)	3.50 (2.16–5.68) < 0.001	2.82 (1.74–4.58) < 0.001	1.13 (0.56–2.25) 0.73
eGDR < 4	11 (3.5)	0.49 (0.20–0.79)	6.06 (3.21–11.46) < 0.001	4.45 (2.35–8.43) < 0.001	0.91 (0.34–2.40) 0.85
Kidney disease					
eGDR ≥ 8	1321 (6.5)	0.92 (0.87–0.97)	REF	REF	REF
6 ≤ eGDR < 8	526 (13.3)	1.53 (1.40–1.66)	1.64 (1.48–1.82) < 0.001	1.53 (1.38–1.69) < 0.001	1.30 (1.11–1.52) 0.001
4 ≤ eGDR < 6	255 (26.8)	3.20 (2.81–3.59)	3.45 (3.01–3.94) < 0.001	3.07 (2.68–3.52) < 0.001	1.58 (1.25–1.99) < 0.001
eGDR < 4	145 (46.6)	5.72 (4.79–6.65)	6.19 (5.21–7.36) < 0.001	5.21 (4.36–6.21) < 0.001	1.33 (0.95–1.86) 0.097
Microalbuminuria vs. non-albuminuria					
eGDR ≥ 8	1251 (6.1)	0.88 (0.83–0.93)	REF	REF	REF
6 ≤ eGDR < 8	489 (12.3)	1.45 (1.32–1.58)	1.62 (1.46–1.80) < 0.001	1.52 (1.37–1.69) < 0.001	1.34 (1.15–1.58) < 0.001
4 ≤ eGDR < 6	227 (23.8)	2.90 (2.52–3.28)	3.25 (2.82–3.75) < 0.001	2.94 (2.55–3.40) < 0.001	1.57 (1.23–1.99) < 0.001
eGDR < 4	129 (41.5)	5.39 (4.46–6.32)	6.08 (5.07–7.30) < 0.001	5.28 (4.38–6.36) < 0.001	1.39 (0.99–1.95) 0.060
Macroalbuminuria vs. non-albuminuria					
eGDR ≥ 8	129 (0.6)	0.09 (0.07–0.10)	REF	REF	REF
6 ≤ eGDR < 8	84 (2.1)	0.23 (0.18–0.28)	2.59 (1.96–3.41) < 0.001	2.14 (1.63–2.83) < 0.001	1.34 (0.91–1.95) 0.13
4 ≤ eGDR < 6	69 (7.3)	0.78 (0.60–0.96)	8.53 (6.35–11.45) < 0.001	6.21 (4.62–8.36) < 0.001	1.61 (0.97–2.70) 0.068
eGDR < 4	39 (12.5)	1.34 (0.92–1.76)	14.57 (10.16–20.89) < 0.001	9.15 (6.34–13.21) < 0.001	1.00 (0.48–2.06) 1.00

*eGDR* estimated glucose disposal rate, *PDR* proliferative diabetic retinopathy, *PPDR* preproliferative diabetic retinopathy

### Comparison of different degrees of retinopathy in relation to eGDR

We further assigned individuals to different degrees of retinopathy to compare the risk for severe retinopathy with milder degrees: PPDR/PRD/laser photocoagulation vs. non/simplex retinopathy and PRD/laser photocoagulation vs. non/simplex retinopathy/PPDR, respectively, into the same eGDR categories (Table 3).

Number of events and event rates was much less for severe retinopathy compared with mild retinopathy. Crude and adjusted for sex and age relative risk for PPDR/PRD/laser photocoagulation vs. non/simplex retinopathy increased in all eGDR categories below 8 mg/kg/min (Table 3). After multivariable adjustments the relative risks, HR (95% CI) for severe retinopathy, i.e. PPDR/PRD/laser photocoagulation vs. non/simplex retinopathy were significantly increased in the eGDR categories 8-6, 6-4, but not in the lowest eGDR group, compared with the reference category (Table 3). Corresponding relative risks after multivariable adjustments between higher degrees of retinopathy, i.e. PRD/laser photocoagulation vs. non/simplex retinopathy/PPDR PRD/laser were not statistical different compared to the reference category (Table 3).

### eGDR and kidney disease

The estimated crude survival curves illustrating the accumulated risk for kidney disease, i.e. microalbuminuria, or macroalbuminuria are shown in Fig. 1B. Median follow up time for the kidney disease cohort was 5.4 (IQR 2.9–8.7) years. The event rate of kidney disease in each eGDR categories, and the relative risks between eGDR and kidney disease are shown in Table 3.

After adjustment for age and sex, and all covariates in Table 1, lower eGDR was associated with an increased risk of kidney disease (Table 3). The adjusted HRs (95% CI) for kidney disease was associated with lower eGDR, however not apply to the lowest category, compared to the reference category (Table 3). The presence of retinopathy was not significant (P = 0.14) when included as a covariate, indicating no competing risk between kidney disease and retinopathy (Additional file 1: Table S4).

### Comparison of different degrees of kidney disease in relation to the eGDR categories

We further assigned individuals to different degrees of kidney disease, i.e. comparison of microalbuminuria and macroalbuminuria vs. non-albuminuria respectively. Number of events and event rates was much less for macroalbuminuria compared with microalbuminuria (Table 3). Crude and adjusted for sex and age relative risk for microalbuminuria and macroalbuminuria, respectively, were increased in all eGDR categories below 8 mg/kg/min (Table 3). After multivariable adjustments the relative risk, HR (95% CI), for microalbuminuria was significantly increased in the eGDR categories 8-6 and 6-4, without reaching statistical significance in the lowest eGDR category, compared to the reference category of eGDR (Table 3). Corresponding relative risks after multivariable adjustments for macroalbuminuria, did not reach any statistical difference between eGDR categories, compared to the reference category of eGDR (Table 3).

### eGDR estimated with ISO-BMI, or BMI, respectively

The reason for using ISO-BMI, instead of BMI, is that this formula “correct” BMI, especially for the youngest children [26]. Since the eGDR formula has not been validated for the use of ISO-BMI, we further investigated our outcome of interest only by BMI. The results for retinopathy did not change much, whereas association for kidney disease somewhat increased for the lowest eGDR. However, there were no large changes between analysis (Additional file 1: Table S5).

### Explained variance of the variables for retinopathy and kidney disease in the eGDR formula

The estimated explained relative risk (R^2^ ± SD) for each variable in the eGDR formula for the risk of retinopathy was highest for HbA1c (0.0242 ± 0.0049), followed by BMI (0.0012 ± 0.0011), and hypertension (0.0006 ± 0.0010). Corresponding explained relative risk for kidney disease was for HbA1c (0.0389 ± 0.0103) followed by BMI (0.0163 ± 0.0064), and hypertension (0.0067 ± 0.0059).

## Discussion

This nationwide, observational study shows that eGDR, a proxy for insulin resistance, associates with the risk of retinopathy and kidney disease in young individuals with T1D. The risk of retinopathy increased with lower eGDR. Risks of kidney disease also increased with lower eGDR, however not for the lowest eGDR category (< 4 mg/kg/min).eGDR has been proven a tool for the measurement of insulin resistance in people with T1D [11] and has emerged as a predictor of cardiovascular complications and mortality [16, 20]. Since microvascular complications often proceed cardiovascular complications [1], it is important to curb the progress of microvascular complications at an early stage to prevent further organ damage and macrovascular complications [33]. Poor glycemic control and hypertension are both well-established risk factors for the development of retinopathy and kidney disease. In the present study, eGDR, as a proxy for insulin resistance, was associated with significantly increased relative risks of retinopathy of different severity and macroalbuminuria in the crude model. However, in the fully adjusted model, the relative risks were no longer statistically significantly increased. This was most likely due to the low number of events of the more severe forms of retinopathy (n = 174 for PDR and laser photocoagulation) as retinopathy develops over time [34], and that our study population was young. For microalbuminuria there was a significantly increased risk with lower eGDR level, except for the lowest eGDR category. Again, this was most likely due to the low number of events in this category.eGDR is calculated from a few clinical measures, which can all individually contribute to our results. In the present study, about a quarter of study participants were overweight (BMI ≥ 25 kg/m^2^) and 9% were obese (BMI ≥ 30 kg/m^2^) according to the World Health Organization’s definition. The prevalence of obesity in childhood T1D populations is increasing. In the Nordic countries the obesity rate was 18.5% in children with T1D under 15 years of age during the period 2008–2012, which was higher than for the Swedish reference population [35]. Risk factors for obesity in this group are longer diabetes duration, higher insulin dose, pump treatment, experiencing frequent severe hypoglycemia, and low HbA1c [35]. We adjusted for several of these covariates in the final model, demonstrating that eGDR might be an important risk factor for microvascular complications in young people with T1D [35, 36].

The impact of obesity and microvascular burden in patients with T1D is not fully established [5]. The prevalence of obesity, i.e. BMI ≥ 30 kg/m^2^, in the population in the DCCT/EDIC study increased from 1 to 31% over the course of 12 years [5]. One possible explanation could be that intensive glycemic control can be associated with weight gain [7, 8, 37]. Another cause might be that obesity is increasing in people with T1D [36, 38], as in the general population. People with T1D and excessive weight gain also have changes in lipid levels and blood pressure similar to those changes seen in insulin resistance syndrome, and a greater central fat distribution [37]. This may contribute to the risk of retinopathy and kidney disease [39]. However, after adjustment for blood lipids there was still an increased risk in people with low eGDR, which was also recently observed by others [23].

Hypertension, often coexist with insulin resistance, is a well-known risk factor for micro- and macrovascular complications [5]. In the current cohort study, the median age was low (21 years) and only 5% had a diagnosis of hypertension and 3% used antihypertensive drugs. The prevalence of hypertension in our study is in line with a previous study on children and adolescents with T1D reporting a rate of hypertension of 5.9% [28]. In the Coronary Artery Calcification (CACTI) study, the prevalence of antihypertensive drugs among individuals with T1D (median age 45 years) was 43% versus 15% in age and sex-matched controls [40]. In the FinnDiane cohort 40% of individuals with T1D were on antihypertensive medication versus 14% of controls [41]. This highlights that the prevalence of hypertension increases with age, also in a population of individuals with T1D even though hypertension might be diagnosed at an earlier age. The low rate of hypertension in our study is probably one of the explanations why most of the study participants had a high eGDR mean 9 mg/kg/min (low insulin resistance). However, in spite of low numbers of hypertension, we found a linear association between eGDR and retinopathy and kidney disease, suggesting eGDR as an important early marker for insulin resistance associated with microvascular complications. In a recent study from our group we demonstrated that early signs of atherosclerosis was associated with increased insulin resistance (measured by clamp technique) in young T1D people without hypertension [42], supporting that risk assessment in people with T1D might include eGDR [16].

Hyperglycemia is a well-known risk factor for both macrovascular and microvascular complications [2, 3]. Hypertension is a well-established risk factor for microvascular complications [43], and treatment of blood pressure decrease the disease progression. There are also data from large cohorts showing that abdominal obesity increases the risk of kidney disease [44, 45]. These three factors are the basis of the eGDR formula and cannot be adjusted for in our model. By using Hellers formula to assess the individual contribution of each variable in the model we simply observed that HbA1c was strongest associated with both retinopathy and kidney disease. It was also recently observed in a cohort of people with T1D, with different age compared to the present cohort study, that eGDR was strongly associated with both microvascular and macrovascular complications, regardless of HbA1c levels [23]. In a recent large cohort study of adult people with T1D, our group show that there was a strong association between eGDR and preterm all-cause mortality [16]. Individuals with eGDR > 8 mg/kg/min had the same expected survival as an age- and sex-matched control group, although HbA1c was 61 mmol/mol [16]. This finding suggests that increased HbA1c is not the sole predictor of micro-, and macrovascular complications and mortality in patients with T1D.

The main strength of this study is the large nationwide population of individuals with T1D with a long follow up. The results show an association between eGDR and risk of retinopathy and kidney disease. We were able to adjust for several important confounders. However, there are limitations to this study. The eGDR formula, including hypertension, has not entirely been validated in youth [46]. Different formula of eGDR may work well as a proxy for insulin resistance [12], but is has also been demonstrated a poor correlation [12–14] between the original eGDR formula by Williams et al. [11], as was used in our study. Although, the eGDR has been shown to be a good marker of increased risk for different outcomes in many cohorts [15–17, 23], we cannot be sure that it really reflects insulin resistance. Despite a long time span there were few individuals with retinopathy, or kidney disease in the lowest eGDR groups during childhood. It was therefore not possible to obtain reliable results in sub-cohort analysis between children and adults. A large number of individuals were excluded (61% in retinopathy cohort and 55% in kidney disease cohort) and we cannot rule out that their prognosis differed from that of the individuals in the study cohort. Most of the excluded individuals had no registration of examination of retinopathy or kidney disease, probably due to new onset of T1D. However, based on the characteristics and risk factors of our excluded cohort it is unlikely that rates of undiagnosed retinopathy and kidney disease were high. Furthermore, there is an inability to adjust for HbA1c as a confounder since it is a part of the eGDR formula, although this formula is created as a proxy for insulin resistance in people with T1D [11]. By using Hellers attributable fraction we demonstrated how much the different components of the eGDR formula contribute to our result, in which HbA1c was the strongest factor in our cohort. Despite this, eGDR, except for the highest HbA1c levels, was associated with retinopathy and kidney disease regardless of HbA1c. Also, there are potential, as in any observational study, known residual confounding factors, i.e. insulin dosing and frequency of hypoglycemia, and unknown residual confounding factors that could have affected our results.

In conclusion, the current cohort study shows that young people with T1D with low eGDR have higher risk of developing both retinopathy and kidney disease, which indicates that insulin resistance increases the risk of these complications. Since microvascular complications are predictors for cardiovascular disease, and earlier observation demonstrates the association between eGDR and cardiovascular death and mortality, in individuals with T1D, further studies are much needed to explore insulin resistance as a risk factor for micro-, and macrovascular complications in people with T1D.

## Supplementary Information

**Additional file 1: Table S1. **Baseline characteristics of all patients divided in the retinopathy and kidney disease cohort and excluded patients, respectively. **Table S2. **Number of measurements and timespan per patient, median and (range). **Table S3. **Baseline characteristics of all patients according to eGDR categories. **Table S4**. Interaction analysis between the retinopathy cohort (10 289 individuals) and the kidney disease cohort (11 857 individuals) with an overlap of 9575 individuals. Number of events, event rate and relative risks for any retinopathy and kidney disease in children and adults with type 1 diabetes. **Table S5**. eGDR based only on BMI (ISO-BMI excluded). Number of events, event rate and relative risks for any retinopathy and kidney disease in children and adults with type 1 diabetes. **Figure S1**. Flowchart for the studied group. The overlap between cohorts included 9575 individuals. Proportion of excluded patients between the registers was for the retinopathy cohort 70% vs. 47% (SwedDiabKid vs. NDR) and for the kidney analysis corresponding proportional numbers were 62% vs. 39% (SwedDiabKid vs. NDR), for the two registers. eGDR, estimated glucose disposal rate; NDR, National Diabetes Register.

## Data Availability

The datasets analysed during the current study are available from the corresponding author on reasonable request.

## References

[1] Avogaro A, Fadini GP (2019). Microvascular complications in diabetes: a growing concern for cardiologists. Int J Cardiol.

[2] Nathan DM, Cleary PA, Backlund JY, Genuth SM, Lachin JM, Orchard TJ, Raskin P, Zinman B, Diabetes C, Complications Trial/Epidemiology of Diabetes I (2005). Intensive diabetes treatment and cardiovascular disease in patients with type 1 diabetes. N Engl J Med.

[3] Writing Team for the Diabetes C, Complications Trial/Epidemiology of Diabetes I, Complications Research G (2003). Sustained effect of intensive treatment of type 1 diabetes mellitus on development and progression of diabetic nephropathy: the Epidemiology of Diabetes Interventions and Complications (EDIC) study. JAMA.

[4] Lind M, Svensson AM, Kosiborod M, Gudbjornsdottir S, Pivodic A, Wedel H, Dahlqvist S, Clements M, Rosengren A (2014). Glycemic control and excess mortality in type 1 diabetes. N Engl J Med.

[5] de Ferranti SD, de Boer IH, Fonseca V, Fox CS, Golden SH, Lavie CJ, Magge SN, Marx N, McGuire DK, Orchard TJ (2014). Type 1 diabetes mellitus and cardiovascular disease: a scientific statement from the American Heart Association and American Diabetes Association. Diabetes Care.

[6] Teupe B, Bergis K (1991). Epidemiological evidence for “double diabetes”. Lancet.

[7] Cleland SJ (2012). Cardiovascular risk in double diabetes mellitus–when two worlds collide. Nat Rev Endocrinol.

[8] Kietsiriroje N, Pearson S, Campbell M, Ariens RAS, Ajjan RA (2019). Double diabetes: a distinct high-risk group?. Diabetes Obes Metab.

[9] Donga E, Dekkers OM, Corssmit EP, Romijn JA (2015). Insulin resistance in patients with type 1 diabetes assessed by glucose clamp studies: systematic review and meta-analysis. Eur J Endocrinol.

[10] DeFronzo RA, Tobin JD, Andres R (1979). Glucose clamp technique: a method for quantifying insulin secretion and resistance. Am J Physiol.

[11] Williams KV, Erbey JR, Becker D, Arslanian S, Orchard TJ (2000). Can clinical factors estimate insulin resistance in type 1 diabetes?. Diabetes.

[12] Duca LM, Maahs DM, Schauer IE, Bergman BC, Nadeau KJ, Bjornstad P, Rewers M, Snell-Bergeon JK (2016). Development and validation of a method to estimate insulin sensitivity in patients with and without type 1 diabetes. J Clin Endocrinol Metab.

[13] Januszewski AS, Sachithanandan N, Ward G, Karschimkus CS, O'Neal DN, Jenkins AJ (2020). Estimated insulin sensitivity in Type 1 diabetes adults using clinical and research biomarkers. Diabetes Res Clin Pract.

[14] Uruska A, Zozulinska-Ziolkiewicz D, Niedzwiecki P, Pietrzak M, Wierusz-Wysocka B (2018). TG/HDL-C ratio and visceral adiposity index may be useful in assessment of insulin resistance in adults with type 1 diabetes in clinical practice. J Clin Lipidol.

[15] Ekstrand AV, Groop PH, Gronhagen-Riska C (1998). Insulin resistance precedes microalbuminuria in patients with insulin-dependent diabetes mellitus. Nephrol Dial Transplant.

[16] Nystrom T, Holzmann MJ, Eliasson B, Svensson AM, Sartipy U (2018). Estimated glucose disposal rate predicts mortality in adults with type 1 diabetes. Diabetes Obes Metab.

[17] Thorn LM, Forsblom C, Fagerudd J, Thomas MC, Pettersson-Fernholm K, Saraheimo M, Waden J, Ronnback M, Rosengard-Barlund M, Bjorkesten CG (2005). Metabolic syndrome in type 1 diabetes: association with diabetic nephropathy and glycemic control (the FinnDiane study). Diabetes Care.

[18] Kilpatrick ES, Rigby AS, Atkin SL (2007). Insulin resistance, the metabolic syndrome, and complication risk in type 1 diabetes: “double diabetes” in the Diabetes Control and Complications Trial. Diabetes Care.

[19] Merger SR, Kerner W, Stadler M, Zeyfang A, Jehle P, Muller-Korbsch M, Holl RW, DPV Initiative, German BCNDm (2016). Prevalence and comorbidities of double diabetes. Diabetes Res Clin Pract.

[20] Orchard TJ, Olson JC, Erbey JR, Williams K, Forrest KY, Smithline Kinder L, Ellis D, Becker DJ (2003). Insulin resistance-related factors, but not glycemia, predict coronary artery disease in type 1 diabetes: 10-year follow-up data from the Pittsburgh Epidemiology of Diabetes Complications Study. Diabetes Care.

[21] Chaturvedi N, Sjoelie AK, Porta M, Aldington SJ, Fuller JH, Songini M, Kohner EM, Study EPC (2001). Markers of insulin resistance are strong risk factors for retinopathy incidence in type 1 diabetes. Diabetes Care.

[22] Girgis CM, Scalley BD, Park KE (2012). Utility of the estimated glucose disposal rate as a marker of microvascular complications in young adults with type 1 diabetes. Diabetes Res Clin Pract.

[23] Helliwell R, Warnes H, Kietsiriroje N, Campbell M, Birch R, Pearson SM, Ajjan RA (2021). Body mass index, estimated glucose disposal rate and vascular complications in type 1 diabetes: beyond glycated haemoglobin. Diabet Med.

[24] Orchard TJ, Chang YF, Ferrell RE, Petro N, Ellis DE (2002). Nephropathy in type 1 diabetes: a manifestation of insulin resistance and multiple genetic susceptibilities? Further evidence from the Pittsburgh Epidemiology of Diabetes Complication Study. Kidney Int.

[25] Eeg-Olofsson K, Cederholm J, Nilsson PM, Zethelius B, Svensson AM, Gudbjornsdottir S, Eliasson B (2010). Glycemic control and cardiovascular disease in 7,454 patients with type 1 diabetes: an observational study from the Swedish National Diabetes Register (NDR). Diabetes Care.

[26] Cole TJ, Bellizzi MC, Flegal KM, Dietz WH (2000). Establishing a standard definition for child overweight and obesity worldwide: international survey. BMJ.

[27] Hoelzel W, Weykamp C, Jeppsson JO, Miedema K, Barr JR, Goodall I, Hoshino T, John WG, Kobold U, Little R (2004). IFCC reference system for measurement of hemoglobin A1c in human blood and the national standardization schemes in the United States, Japan, and Sweden: a method-comparison study. Clin Chem.

[28] Rodriguez BL, Dabelea D, Liese AD, Fujimoto W, Waitzfelder B, Liu L, Bell R, Talton J, Snively BM, Kershnar A (2010). Prevalence and correlates of elevated blood pressure in youth with diabetes mellitus: the SEARCH for diabetes in youth study. J Pediatr.

[29] Tsai WY, Jewell NP, Wang MC (1987). A note on the product-limit estimator under right censoring and left truncation. Biometrika.

[30] Goodall RL, Dunn DT, Babiker AG (2004). Interval-censored survival time data: confidence intervals for the non-parametric survivor function. Stat Med.

[31] Snapinn SM, Jiang Q, Iglewicz B (2005). Illustrating the impact of a time-varying covariate with an extended Kaplan-Meier estimator. Am Stat.

[32] Heller G (2012). A measure of explained risk in the proportional hazards model. Biostatistics.

[33] Rosenson RS, Fioretto P, Dodson PM (2011). Does microvascular disease predict macrovascular events in type 2 diabetes?. Atherosclerosis.

[34] Hietala K, Harjutsalo V, Forsblom C, Summanen P, Groop PH, FinnDiane Study G (2010). Age at onset and the risk of proliferative retinopathy in type 1 diabetes. Diabetes Care.

[35] Birkebaek NH, Kahlert J, Bjarnason R, Drivvoll AK, Johansen A, Konradsdottir E, Pundziute-Lycka A, Samuelsson U, Skrivarhaug T, Svensson J (2018). Body mass index standard deviation score and obesity in children with type 1 diabetes in the Nordic countries. HbA(1c) and other predictors of increasing BMISDS. Pediatr Diabetes.

[36] Vilarrasa N, San Jose P, Rubio MA, Lecube A (2021). Obesity in patients with type 1 diabetes: links, risks and management challenges. Diabetes Metab Syndr Obes.

[37] Purnell JQ, Zinman B, Brunzell JD, Group DER (2013). The effect of excess weight gain with intensive diabetes mellitus treatment on cardiovascular disease risk factors and atherosclerosis in type 1 diabetes mellitus: results from the Diabetes Control and Complications Trial/Epidemiology of Diabetes Interventions and Complications Study (DCCT/EDIC) study. Circulation.

[38] Stokes A, Ni Y, Preston SH (2017). Prevalence and trends in lifetime obesity in the U.S., 1988–2014. Am J Prev Med.

[39] Rathsman B, Haas J, Persson M, Ludvigsson J, Svensson AM, Lind M, Andersson Franko M, Nystrom T (2021). LDL cholesterol level as a risk factor for retinopathy and nephropathy in children and adults with type 1 diabetes mellitus: a nationwide cohort study. J Intern Med.

[40] Schauer IE, Snell-Bergeon JK, Bergman BC, Maahs DM, Kretowski A, Eckel RH, Rewers M (2011). Insulin resistance, defective insulin-mediated fatty acid suppression, and coronary artery calcification in subjects with and without type 1 diabetes: The CACTI study. Diabetes.

[41] Ronnback M, Fagerudd J, Forsblom C, Pettersson-Fernholm K, Reunanen A, Groop PH, Finnish Diabetic Nephropathy Study (2004). Altered age-related blood pressure pattern in type 1 diabetes. Circulation.

[42] Rathsman B, Rosfors S, Sjoholm A, Nystrom T (2012). Early signs of atherosclerosis are associated with insulin resistance in non-obese adolescent and young adults with type 1 diabetes. Cardiovasc Diabetol.

[43] Colhoun HM, Lee ET, Bennett PH, Lu M, Keen H, Wang SL, Stevens LK, Fuller JH (2001). Risk factors for renal failure: the WHO Mulinational Study of Vascular Disease in Diabetes. Diabetologia.

[44] Chaturvedi N, Bandinelli S, Mangili R, Penno G, Rottiers RE, Fuller JH (2001). Microalbuminuria in type 1 diabetes: rates, risk factors and glycemic threshold. Kidney Int.

[45] de Boer IH, Sibley SD, Kestenbaum B, Sampson JN, Young B, Cleary PA, Steffes MW, Weiss NS, Brunzell JD, Diabetes C (2007). Central obesity, incident microalbuminuria, and change in creatinine clearance in the epidemiology of diabetes interventions and complications study. J Am Soc Nephrol.

[46] Dabelea D, D'Agostino RB, Mason CC, West N, Hamman RF, Mayer-Davis EJ, Maahs D, Klingensmith G, Knowler WC, Nadeau K (2011). Development, validation and use of an insulin sensitivity score in youths with diabetes: the SEARCH for Diabetes in Youth study. Diabetologia.

