# Supplementary material for: Estimated glucose disposal rate is associated with retinopathy and kidney disease in young people with type 1 diabetes: a nationwide observational study
Source: Cardiovasc Diabetol. 2023 Mar 19;22:61. doi: 10.1186/s12933-023-01791-x (PMC10024828; doi:10.1186/s12933-023-01791-x)
Supplement: Supplementary file 1 — Additional file 1: Table S1. Baseline characteristics of all patients divided in the retinopathy and kidney disease cohort and excluded patients, respectively. Table S2. Number of measurements and timespan per patient, median and (range). Table S3. Baseline characteristics of all patients according to eGDR categories. Table S4. Interaction analysis between the retinopathy cohort (10 289 individuals) and the kidney disease cohort (11 857 individuals) with an overlap of 9575 individuals. Number of events, event rate and relative risks for any retinopathy and kidney disease in children and adults with type 1 diabetes. Table S5. eGDR based only on BMI (ISO-BMI excluded). Number of events, event rate and relative risks for any retinopathy and kidney disease in children and adults with type 1 diabetes. Figure S1. Flowchart for the studied group. The overlap between cohorts included 9575 individuals. Proportion of excluded patients between the registers was for the retinopathy cohort 70% vs. 47% (SwedDiabKid vs. NDR) and for the kidney analysis corresponding proportional numbers were 62% vs. 39% (SwedDiabKid vs. NDR), for the two registers. eGDR, estimated glucose disposal rate; NDR, National Diabetes Register. [file 12933_2023_1791_MOESM1_ESM.docx]

# **Additional file**

# **Estimated glucose disposal rate is associated with retinopathy and kidney disease in young people with type 1 diabetes: a nationwide observational study**

Wedén Linn^1^, Martina Persson^2^, Björn Rathsman^2^, Johnny Ludvigsson^3,4^, Marcus Lind^5,6^, Mikael Andersson Franko^1^, Thomas Nyström^1^

^1^Department of Clinical Science and Education, Karolinska Institutet, Södersjukhuset, Stockholm, Sweden

^2^Sachs’ Children and Youth Hospital, Södersjukhuset, Stockholm, Sweden

^3^Division of Paediatrics, Department of Biomedical and Clinical Sciences, Linköping University, Linköping, Sweden

^4^Crown Princess Victoria Children’s Hospital, Region Östergötland, Linköping, Sweden

^5^Department of Medicine, NU Hospital Group, Uddevalla, Sweden

^6^ Department of Molecular and Clinical Medicine, Institute of Medicine, University of Gothenburg

**Table S1.** Baseline characteristics of all patients divided in the retinopathy and kidney disease cohort and excluded patients, respectively.

|  | Excluded patients^**^ | Retinopathy analysis | p-value | Kidney disease analysis | p-value |
| --- | --- | --- | --- | --- | --- |
| Number | 14214 | 10289 |  | 11857 |  |
| Age, yrs | 12 (7 – 22) | 21 (19 – 26) | <0.001 | 21 (19 – 26) | <0.001 |
| Males | 55.3% | 58.1% | <0.001 | 57.3% | 0.002 |
| Debut age, yrs | 11 (6 – 17) | 16 (11 – 21) | <0.001 | 15 (10 – 21) | <0.001 |
| 0-10 yrs | 44.8% | 18.3% | <0.001 | 22.9% | <0.001 |
| 10-15 yrs | 25.5% | 26.3% |  | 25.7% |  |
| 15-20 yrs | 11.4% | 23.7% |  | 21.9% |  |
| 20-25 yrs | 10.7% | 18.9% |  | 17.6% |  |
| 25-30 yrs | 7.6% | 12.7% |  | 11.9% |  |
| Duration, yrs | 0.2 (0.1 – 5) | 6 (3 – 9) | 0.27 | 7 (3 – 10) | 0.046 |
| Follow-up time, yrs | 3.4 (1.0 – 7.2) | 4.8 (2.6 – 7.7) | <0.001 | 5.4 (2.9 – 8.7) | <0.001 |
| eGDR measurements per individual | 8 (2 – 21) | 4 (2 – 6) | <0.001 | 4 (2 – 7) | <0.001 |
| eGDR | 9.3 (8.3 – 10.0) | 9.0 (8.0 – 9.8) | 0.40 | 8.9 (8.0 – 9.7) | 0.25 |
| < 4 | 1.3% | 1.7% | 0.37 | 1.5% | 0.15 |
| 4 ≤ to < 6 | 3.6% | 5.1% |  | 5.0% |  |
| 6 ≤ to < 8 | 15.2% | 18.5% |  | 19.4% |  |
| ≥ 8 | 80.0% | 74.8% |  | 74.1% |  |
| LDL-cholesterol, mmol/L | 2.37 (1.90 – 2.90) | 2.43 (1.97 – 2.96) | 0.003 | 2.43 (1.98 – 2.98) | <0.001 |
| < 2.6 | 61.7% | 59.1% | 0.25 | 58.6% | 0.066 |
| 2.6 ≤ to < 3.4 | 26.6% | 28.3% |  | 28.3% |  |
| 3.4 ≤ to < 4.1 | 8.1% | 8.9% |  | 9.3% |  |
| ≥ 4.1 | 3.5% | 3.6% |  | 3.8% |  |
| HDL-cholesterol, mmol/L | 1.5 (1.2 – 1.8) | 1.4 (1.2 – 1.7) | <0.001 | 1.4 (1.2 – 1.7) | <0.001 |
| < 1.1 | 11.9% | 13.1% | <0.001 | 13.2% | <0.001 |
| ≥ 1.1 | 88.1% | 86.9% |  | 86.8% |  |
| Total-cholesterol, mmol/L | 4.3 (3.8 – 4.9) | 4.4 (3.8 – 5.0) | 0.21 | 4.4 (3.8 – 5.0) | 0.85 |
| < 4.5 | 55.5% | 54.5% | 0.62 | 53.9% | 0.56 |
| ≥ 4.5 | 44.5% | 45.5% |  | 46.1% |  |
| Triglyceride, mmol/L | 0.85 (0.60 – 1.30) | 0.90 (0.62 – 1.28) | 0.13 | 0.90 (0.66 – 1.30) | 0.007 |
| < 1.7 | 85.9% | 86.5% | 0.21 | 86.1% | 0.43 |
| ≥ 1.7 | 14.1% | 13.5% |  | 13.9% |  |
| HbA1c, mmol/mol | 59 (49 – 71) | 60 (51 – 71) | 0.11 | 61 (52 – 72) | 0.21 |
| < 48 | 20.3% | 17.2% | <0.001 | 16.4% | <0.001 |
| 48 ≤ to < 58 | 26.6% | 24.9% |  | 23.8% |  |
| 58 ≤ to < 70 | 25.3% | 29.7% |  | 30.5% |  |
| ≥ 70 | 27.8% | 28.2% |  | 29.2% |  |
| BMI (Kg/m^2*^) | 23.0 (20.9 – 25.6) | 23.6 (21.6 – 26.2) | <0.001 | 23.4 (21.3 – 26.1) | <0.001 |
| Normal | 70.3% | 64.5% | <0.001 | 65.4% | <0.001 |
| Overweight | 21.6% | 26.3% |  | 25.7% |  |
| Obese | 8.1% | 9.2% |  | 8.9% |  |
| Smokers | 6.8% | 11.0% | <0.001 | 11.3% | <0.001 |
| Physical activity; daily | 21.9% | 18.2% | <0.001 | 17.6% | <0.001 |
| Physical activity; 3-5 times/week | 31.5% | 33.2% |  | 33.6% |  |
| Physical activity; 1-2 times/week | 31.6% | 26.2% |  | 26.9% |  |
| Physical activity; <1 times/week | 8.4% | 14.1% |  | 13.7% |  |
| Physical activity; never | 6.6% | 8.3% |  | 8.1% |  |
| Insulin method; injection | 81.7% | 81.3% | <0.001 | 79.5% | 0.47 |
| Insulin method; pump | 18.3% | 18.7% |  | 20.5% |  |
| ASA; yes | 1.1% | 0.8% | <0.001 | 0.7% | <0.001 |
| Antihypertensive; yes | 4.3% | 3.5% | <0.001 | 3.0% | <0.001 |
| Lipid lowering drug; yes | 2.5% | 3.0% | <0.001 | 2.6% | <0.001 |
| Hypertension | 2.8% | 5.2% | <0.001 | 5.0% | <0.001 |
| eGFR, ml/min | 127 (104 – 160) | 123 (106 – 145) | <0.001 | 124 (107 – 147) | 0.027 |
| < 30 | 0.13% | 0.06% | <0.001 | 0.03% | <0.001 |
| 30 ≤ to < 45 | 0.10% | 0.03% |  | 0.02% |  |
| 45 ≤ to < 60 | 0.21% | 0.13% |  | 0.10% |  |
| 60 ≤ to < 90 | 10.92% | 7.26% |  | 6.79% |  |
| ≥ 90 | 88.63% | 92.53% |  | 93.06% |  |

ASA, Acetylsalicylic acid; BMI, Body mass index; eGFR, Estimated glomerular filtration rate; HbA1c, Glycated hemoglobin 1c; HDL-Cholesterol, High-density lipoprotein-Cholesterol; LDL-Cholesterol, Low-density lipoprotein-Cholesterol. Yrs, years.^*^isoBMI was used in individuals <18 yrs. ^**^Proportion of excluded patients between the registers was for the retinopathy cohort 70% vs. 47% (SwedDiabKid vs NDR) and for the kidney analysis corresponding proportional numbers were 62% vs. 39% (SwedDiabKid vs NDR), for the two registers.

# **Table S2.** Number of measurements and timespan per patient, median and (range)

|  | Number of measurements | Timespan |
| --- | --- | --- |
| LDL-cholesterol, mmol/L | 2 (0 – 65) | 0.9 (0.0 – 15.2) |
| HDL-cholesterol, mmol/L | 2 (0 – 66) | 0.9 (0.0 – 17.2) |
| Total-cholesterol, mmol/L | 2 (0 – 66) | 1.1 (0.0 – 17.7) |
| Triglyceride, mmol/L | 2 (0 – 66) | 0.5 (0.0 – 17.3) |
| HbA1c, mmol/mol | 12 (0 – 124) | 5.7 (0.0 – 18.0) |
| BMI (Kg/m^2*^) | 10 (0 – 122) | 5.2 (0.0 – 17.9) |
| Smokers | 7 (0 – 78) | 4.3 (0.0 – 17.9) |
| Physical activity | 6 (0 – 74) | 3.2 (0.0 – 13.6) |
| Insulin method | 11 (0 – 124) | 4.9 (0.0 – 17.9) |
| ASA | 2 (0 – 72) | 1.0 (0.0 – 15.2) |
| Antihypertensive | 3 (0 – 72) | 1.5 (0.0 – 16.4) |
| Lipid lowering drug | 3 (0 – 72) | 1.5 (0.0 – 16.4) |
| Hypertension | 13 (1 – 131) | 5.8 (0.0 – 18.0) |
| eGFR, ml/min | 2 (0 – 69) | 0.8 (0.0 – 17.7) |

ASA, Acetylsalicylic acid; BMI, Body mass index; eGFR, Estimated glomerular filtration rate; HbA1c, Glycated hemoglobin 1c; HDL-Cholesterol, High-density lipoprotein-Cholesterol; LDL-Cholesterol, Low-density lipoprotein-Cholesterol. Yrs, years. ^*^isoBMI was used in individuals <18 yrs.

# **Table S3.** Baseline characteristics of all patients according to eGDR categories.

|  | Total | <4 | 4 to 5.99 | 6 to 7.99 | ≥8 | % missing |
| --- | --- | --- | --- | --- | --- | --- |
| Number | 26785 | 311 | 952 | 3967 | 20407 | 4% |
| Age, yrs | 15 (9–23) | 28 (22 – 31) | 23 (18 – 28) | 19 (11 – 25) | 14 (9 – 21) | - |
| Males | 56.2% | 52.1% | 55.1% | 55.1% | 56.7% | - |
| Debut age, yrs | 13 (8–19) | 23 (18 – 26) | 20 (13 – 24) | 14 (9 – 21) | 12 (7 – 18) | - |
| 0-10 yrs | 34.6% | 5.8% | 12.0% | 28.0% | 38.6% |  |
| 10-15 yrs | 25.7% | 10.3% | 18.4% | 26.8% | 26.3% |  |
| 15-20 yrs | 16.3% | 15.8% | 21.1% | 16.8% | 15.8% |  |
| 20-25 yrs | 13.9% | 32.2% | 27.5% | 16.4% | 11.7% |  |
| 25-30 yrs | 9.6% | 36.0% | 21.0% | 12.0% | 7.6% |  |
| Duration, yrs | 0.3 (0.1–4.0) | 4 (1 – 7) | 2 (0 – 7) | 1 (0 – 6) | 0.2 (0.1 – 3.0) | - |
| Follow-up time, yrs | 5.8 (2.2–9.9) | 2.0 (0.1–5.1) | 3.3 (1.1–7.1) | 5.2 (1.8–9.5) | 6.6 (2.9–10.3) | - |
| eGDR measurements per individual | 10 (3–23) | 3 (1 – 5) | 4 (2 – 9) | 7 (3 – 21) | 12 (5 – 25) | - |
| LDL-cholesterol, mmol/L | 2.40 (1.9-2.9) | 3.00 (2.3-3.4) | 2.76 (2.2-3.4) | 2.59 (2.1-3.2) | 2.34 (1.9-2.8) | 32% |
| < 2.6 | 60.3% | 36.8% | 42.3% | 50.1% | 63.9% |  |
| 2.6 ≤ to < 3.4 | 27.5% | 35.9% | 30.1% | 31.7% | 26.3% |  |
| 3.4 ≤ to < 4.1 | 8.6% | 13.4% | 19.1% | 12.4% | 7.1% |  |
| ≥ 4.1 | 3.6% | 13.9% | 8.5% | 5.8% | 2.7% |  |
| HDL-cholesterol, mmol/L | 1.4 (1.2–1.7) | 1.1 (0.9–1.4) | 1.2 (1.0–1.5) | 1.4 (1.1–1.6) | 1.5 (1.2–1.8) | 31% |
| < 1.1 | 13.4% | 41.8% | 28.6% | 18.0% | 11.0% |  |
| ≥ 1.1 | 86.6% | 58.2% | 71.4% | 82.0% | 89.0% |  |
| Total-cholesterol, mmol/L | 4.3 (3.8 – 4.9) | 5.0 (4.4 – 5.8) | 4.8 (4.1 – 5.5) | 4.5 (3.9 – 5.2) | 4.3 (3.8 – 4.8) | 29% |
| < 4.5 | 55.5% | 29.1% | 38.8% | 46.2% | 58.9% |  |
| ≥ 4.5 | 44.5% | 70.9% | 61.2% | 53.8% | 41.1% |  |
| Triglyceride, mmol/L | 0.9 (0.6-1.3) | 1.9 (1.0-2.8) | 1.2 (0.9-2.1) | 1.0 (0.7-1.5) | 0.8 (0.6-1.2) | 34% |
| < 1.7 | 86.1% | 43.5% | 65.9% | 78.3% | 89.6% |  |
| ≥ 1.7 | 13.9% | 56.5% | 34.1% | 21.7% | 10.4% |  |
| HbA1c, mmol/mol | 59 (49 – 72) | 80 (66 – 102) | 80 (64 – 110) | 78 (65 – 94) | 56 (47 – 66) | 1% |
| < 48 | 21.1% | 3.5% | 5.6% | 6.0% | 25.0% |  |
| 48 ≤ to < 58 | 25.0% | 10.3% | 10.4% | 8.8% | 29.3% |  |
| 58 ≤ to < 70 | 24.7% | 17.4% | 18.4% | 18.6% | 26.4% |  |
| ≥ 70 | 29.1% | 68.8% | 65.7% | 66.6% | 19.2% |  |
| BMI (Kg/m^2*^) | 20.3  (17.3-23.9) | 34.7  (30.8-40.5) | 27.5  (23.1-33.0) | 24.0  (20.2-28.2) | 19.4  (16.9-22.7) | 4% |
| Normal | 80.7% | 8.4% | 34.9% | 56.5% | 88.7% |  |
| Overweight | 13.5% | 12.9% | 26.1% | 26.7% | 10.4% |  |
| Obese | 5.8% | 78.8% | 39.1% | 16.8% | 0.9% |  |
| Smokers | 8.0% | 20.2% | 17.5% | 13.4% | 6.0% | 7% |
| Physical activity; daily | 19.6% | 19.7% | 18.6% | 17.9% | 19.9% | 19% |
| Physical activity; 3-5 times/week | 32.6% | 23.6% | 25.3% | 28.1% | 33.9% |  |
| Physical activity; 1-2 times/week | 29.9% | 27.9% | 29.1% | 30.0% | 30.1% |  |
| Physical activity; <1 times/week | 10.7% | 16.2% | 15.4% | 13.4% | 9.9% |  |
| Physical activity; never | 7.2% | 12.7% | 11.5% | 10.6% | 6.1% |  |
| Insulin method; injection | 82.0% | 96.3% | 91.6% | 83.2% | 80.8% | 8% |
| Insulin method; pump | 18.0% | 3.7% | 8.4% | 16.8% | 19.2% |  |
| ASA; yes | 0.7% | 5.4% | 2.2% | 0.7% | 0.5% | 35% |
| Antihypertensive therapy; yes | 3.0% | 37.5% | 17.9% | 4.0% | 1.0% | 30% |
| Lipid lowering drug; yes | 1.8% | 17.9% | 7.8% | 2.7% | 0.9% | 30% |
| Hypertension | 2.7% | 57.6% | 28.0% | 4.1% | 0.1% | - |
| eGFR, ml/min | 126  (106–152) | 136  (110 – 168) | 131  (106 – 158) | 128  (108 – 155) | 126  (106 – 151) | 35% |
| < 30 | 0.08% | 0.79% | 0.82% | 0.00% | 0.05% |  |
| 30 ≤ to < 45 | 0.05% | 0.00% | 0.54% | 0.07% | 0.02% |  |
| 45 ≤ to < 60 | 0.14% | 1.19% | 0.41% | 0.14% | 0.10% |  |
| 60 ≤ to < 90 | 8.74% | 10.32% | 8.71% | 9.09% | 8.59% |  |
| ≥ 90 | 90.98% | 87.70% | 89.52% | 90.69% | 91.24% |  |

ASA, Acetylsalicylic acid; BMI, Body mass index; eGFR, Estimated glomerular filtration rate; HbA1c, Glycated hemoglobin 1c; HDL-Cholesterol, High-density lipoprotein-Cholesterol; LDL-Cholesterol, Low-density lipoprotein-Cholesterol. Yrs, years.^*^isoBMI was used in individuals <18 yrs.

# **Table S4**. Interaction analysis between the retinopathy cohort (10 289 individuals) and the kidney disease cohort (11 857 individuals) with an overlap of 9575 individuals. Number of events, event rate and relative risks for any retinopathy and kidney disease in children and adults with type 1 diabetes.

| Exposure | Events  n | Event rate  100  person-yrs | Crude HR | HR adjusted for sex and age | HR adjusted |
| --- | --- | --- | --- | --- | --- |
| Retinopathy | | | | | |
| eGDR ≥ 8 | 5040 | 6.92 (6.74 – 7.10) | REF | REF | REF |
| 6 ≤ eGDR < 8 | 1909 | 11.40 (10.89 – 11.91) | 1.45 (1.36 – 1.54)  <0.001 | 1.40 (1.31 – 1.49)  <0.001 | 1.28 (1.18 – 1.38)  <0.001 |
| 4 ≤ eGDR < 6 | 504 | 13.78 (12.58 – 14.99) | 1.65 (1.49 – 1.82)  <0.001 | 1.51 (1.37 – 1.68)  <0.001 | 1.46 (1.27 – 1.68)  <0.001 |
| eGDR < 4 | 179 | 16.85 (14.38 – 19.32) | 1.95 (1.66 – 2.29)  <0.001 | 1.71 (1.45 – 2.00)  <0.001 | 1.70 (1.38 – 2.11)  <0.001 |
| Kidney disease |  |  | 1.18 (1.08 – 1.29)  <0.001 | 1.17 (1.07 – 1.28)  0.001 | 1.07 (0.96 – 1.20)  0.21 |
| Kidney disease | | | | | |
| eGDR ≥ 8 | 1321 | 1.82 (1.72 – 1.92) | REF | REF | REF |
| 6 ≤ eGDR < 8 | 526 | 2.73 (2.50 – 2.97) | 1.60 (1.42 – 1.80)  <0.001 | 1.55 (1.38 – 1.75)  <0.001 | 1.29 (1.10 – 1.51)  0.002 |
| 4 ≤ eGDR < 6 | 255 | 5.58 (4.89 – 6.26) | 3.23 (2.76 – 3.77)  <0.001 | 3.09 (2.64 – 3.61)  <0.001 | 1.52 (1.19 – 1.92)  0.001 |
| eGDR < 4 | 145 | 9.86 (8.26 – 11.47) | 5.35 (4.36 – 6.55)  <0.001 | 4.99 (4.06 – 6.14)  <0.001 | 1.34 (0.95 – 1.90)  0.095 |
| Retinopathy |  |  | 1.11 (1.00 – 1.25)  0.057 | 1.12 (1.00 – 1.25)  0.052 | 1.12 (0.96 – 1.29)  0.14 |

HR; Hazard ratio. PPDR; Preproliferative Diabetes Retinopathy. PDR; Proliferative Diabetes Retinopathy. ^*^Defined as any of; Simplex Retinopathy/PPDR/PDR/Laser. ^**^Defined as any of microalbuminuria or macroalbuminuria.

# **Table S5**. eGDR based only on BMI (ISO-BMI excluded). Number of events, event rate and relative risks for any retinopathy and kidney disease in children and adults with type 1 diabetes.

| Exposure  mmol/L | Events  n | Event rate  100  person-yrs | Crude HR | HR adjusted for sex and age | HR adjusted |
| --- | --- | --- | --- | --- | --- |
| Retinopathy | | | | | |
| eGDR ≥ 8 | 5194 | 4.09 (3.97 – 4.20) | REF | REF | REF |
| 6 ≤ eGDR < 8 | 1775 | 8.25 (7.87 – 8.64) | 1.71 (1.62 – 1.81)  <0.001 | 1.47 (1.39 – 1.56)  <0.001 | 1.28 (1.18 – 1.38)  <0.001 |
| 4 ≤ eGDR < 6 | 488 | 8.97 (8.17 – 9.77) | 1.91 (1.74 – 2.10)  <0.001 | 1.54 (1.40 – 1.69)  <0.001 | 1.44 (1.26 – 1.65)  <0.001 |
| eGDR < 4 | 176 | 9.50 (8.09 – 10.90) | 2.26 (1.94 – 2.63)  <0.001 | 1.70 (1.45 – 1.98)  <0.001 | 1.60 (1.29 – 1.98)  <0.001 |
| Kidney disease | | | | | |
| eGDR ≥ 8 | 1349 | 0.91 (0.86 – 0.95) | REF | REF | REF |
| 6 ≤ eGDR < 8 | 494 | 1.65 (1.50 – 1.79) | 1.79 (1.61 – 1.99)  <0.001 | 1.61 (1.45 – 1.79)  <0.001 | 1.42 (1.22 – 1.66)  <0.001 |
| 4 ≤ eGDR < 6 | 254 | 3.33 (2.92 – 3.73) | 3.63 (3.17 – 4.15)  <0.001 | 3.18 (2.77 – 3.65)  <0.001 | 1.65 (1.31 – 2.08)  <0.001 |
| eGDR < 4 | 151 | 6.10 (5.13 – 7.07) | 6.70 (5.66 – 7.93)  <0.001 | 5.67 (4.77 – 6.75)  <0.001 | 1.60 (1.14 – 2.23)  0.006 |

HR; Hazard ratio. PPDR; Preproliferative Diabetes Retinopathy. PDR; Proliferative Diabetes Retinopathy. ^*^Defined as any of; Simplex Retinopathy/PPDR/PDR/Laser. ^**^Defined as any of microalbuminuria or macroalbuminuria.

**Figure S1**. Flowchart for the studied group. The overlap between cohorts included 9575 individuals. Proportion of excluded patients between the registers was for the retinopathy cohort 70% vs. 47% (SwedDiabKid vs. NDR) and for the kidney analysis corresponding proportional numbers were 62% vs. 39% (SwedDiabKid vs. NDR), for the two registers. eGDR, estimated glucose disposal rate; NDR, National Diabetes Register.

NDR

1998-2017

n=19 298

SWEDIABKIDS

2000-2017

n=15 111

Cases with at least one

micro-, or macroalbuminuria observation

n=22 951

Cases with at least one observation on each covariate

n=10 289

Cases with at least one observation on each covariate

n=11 857

Cases with at least one

eGDR observation

n=21 966

Cases with at least one

eGDR observation

n=20 146

Cases with at least one retinopathy screening examination

n=20 716

SWEDIABKIDS + NDR

Type 1 diabetes with < 10 years duration

n=26 786
